# *Towards Clinical Translation*: Optimized Fabrication of Controlled Nanostructures on Implant-Relevant Curved Zirconium Surfaces

**DOI:** 10.3390/nano11040868

**Published:** 2021-03-29

**Authors:** Divya Chopra, Karan Gulati, Sašo Ivanovski

**Affiliations:** The University of Queensland, School of Dentistry, Herston, QLD 4006, Australia; d.chopra@uq.net.au

**Keywords:** zirconium, zirconia, dental implants, nanopores, electrochemical anodization

## Abstract

Anodization enables fabrication of controlled nanotopographies on Ti implants to offer tailorable bioactivity and local therapy. However, anodization of Zr implants to fabricate ZrO_2_ nanostructures remains underexplored and are limited to the modification of easy-to-manage flat Zr foils, which do not represent the shape of clinically used implants. In this pioneering study, we report extensive optimization of various nanostructures on implant-relevant micro-rough Zr curved surfaces, bringing this technology closer to clinical translation. Further, we explore the use of sonication to remove the top nanoporous layer to reveal the underlying nanotubes. Nano-engineered Zr surfaces can be applied towards enhancing the bioactivity and therapeutic potential of conventional Zr-based implants.

## 1. Introduction

Zirconium (Zr) is a valve metal that is very stable with a high dielectric constant, and hence it is a suitable material choice for the nuclear and microelectronic industries [1]. Further, Zr and its alloys are extensively used in the field of optics, magnetics, chemical sensors, and biomedical implants [2]. Due to their favourable characteristics (physical, chemical, and biological), Zr-based implants are gaining popularity in the dental and orthopaedic markets [3,4]. For this application, the favourable biocompatibility of Zr is mainly attributed to its surface oxide film (ZrO_2_). Further, Zr (metal, grey colour) with a ZrO_2_ (ceramic, white colour) surface has a toughness comparable to metals, and hence is suitable for a variety of biomedical applications [5]. Clinically, ceramic structures have shown a higher risk of fracture due to the nature of the material. However, oxidized Zr surfaces offer the potential to decrease wear and tear as the bulk of the material is metal, and not a monolithic ceramic [6]. It is noteworthy that oxidized Zr is not a ceramic but the transition of metal to ceramic. Studies have established that ZrO_2_ not only promotes osseointegration but also demonstrates reduced cytotoxicity as compared to Ti-based implants [2]. Moreover, ZrO_2_/Zr presents greater mechanical strength and low ion release when compared to Ti [7]. Overall, as compared to Ti, ZrO_2_ based implants offers many advantages including superior aesthetics, with favourable biological, mechanical, and optical properties [8].

In the last few decades, the potential of Zr and its alloys in the field of dental implants has gained increasing attention [9,10,11]. Although there are some in vivo studies that demonstrate the biocompatibility of Zr, the surface modification and related bioactivity assessment of Zr-based implants needs in-depth investigation [2]. It is noteworthy that in compromised patient conditions (e.g., diabetic and osteoporotic), ‘normal’ bioactivity may not be sufficient to encourage bone-implant integration, and hence enhanced bioactivity is needed. In that light, surface modifications of Zr-based implants to form an oxide layer have been performed via various physical, chemical, and electrochemical means [12,13,14]. Further, electrochemical anodization (EA) has been regarded as an effective strategy to fabricate ZrO_2_ with nanoscale surface roughness and the ability to incorporate bioactive ions (Ca or P) [15].

It is well established that the bioactivity of modified implant surfaces follows the trend nano- > micro- > macro-scale [16]. As a result, research has shifted towards the fabrication of controlled nanotopographies on Zr-based implants (including Zr, Ti–Zr alloys etc.). Various strategies have been employed for nano-engineering Zr implants, such as electrochemical anodization (EA) [17], plasma treatment [18], micro-arc oxidation [19], hydrothermal treatment [20], chemical co-precipitation, and sol–gel method [21]. Among these, EA stands out due to its cost-effectiveness, scalability, and control over the characteristics of the fabricated nanostructures [22]. Briefly, EA involves immersion of a target substrate (Zr) as an anode and a counter electrode (cathode) in a suitable electrolyte (containing water and fluoride ions), and a supply of constant voltage/current. Upon attainment of optimized conditions, self-ordering of ZrO_2_ nanotubes occurs on the surface of the anode. Relevant to biomedical applications, EA to fabricate self-ordered ZrO_2_ nanotubes has gained attention, with various attempts made to optimize the EA fabrication [23,24,25]. Further, the augmented bioactivity and osteogenic ability of ZrO_2_ nanotubes has also been demonstrated [26,27,28].

With respect to anodized nano-engineered zirconium implants, key fabrication challenges remain unaddressed:

1. Fabrication optimization has only been restricted to planar Zr flat foil that is easy to manage. However, clinically used orthopaedic and dental implants are based on curved surfaces and edges, thereby limiting the clinical translation of conventional anodized Zr flat foil.
2. Dental implants generally use microscale roughness which, to date, is regarded as a ‘gold standard’ for ensuring osseointegration. Thus, preserving rather than removal of this micro-roughness (which is routinely performed to fabricate nanotubes) is needed along with superimposition of nanostructures (dual micro–nano).

**Figure 1:**
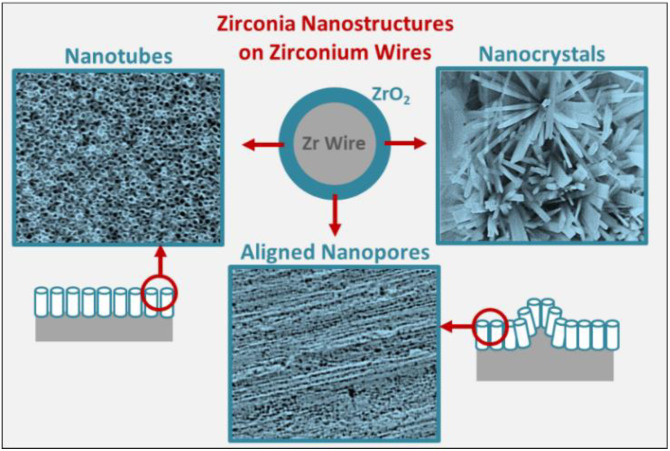
Schematic representation of various nanostructures fabricated on clinical implant relevant zirconium wire.

To further optimize the fabrication of anodic nanostructures on Zr-based implants, in this study, we explore EA optimization of Zr wires as models for curved clinically relevant implant architectures. Briefly, EA parameters, including voltage and time, were varied to fabricate oxide nanocrystals, nanopores, and nanotubes on the Zr wires (Figure 1). This study bridges the gap between the fabrication of controlled nanostructures on clinically relevant Zr surfaces, with the objective of facilitating future clinical translation. We also report on the use of sonication to reveal the underlying nanostructures by removing the superficial nanoporous oxide film. Optimized fabrication of controlled nanotopographies on implant substrates that preserves the underlying micro-roughness can be paradigm shifting in the domain of Zr-based biomedical applications.

## 2. Experimental Section

### 2.1. Materials and Chemicals

Zirconium wire with 0.5 mm diameter [annealed, 99.2% purity (metal basis excluding Hf), 4.5% Hf max] was obtained from Alfa Aesar (Lancashire, UK) and used as received. High-purity (NH_4_)_2_SO_4_, NH_4_F, and methanol were purchased from Sigma Aldrich (North Ryde, Australia).

### 2.2. Electrochemical Anodization (EA)

Prior to EA, as-received Zr wires were cut into 10 cm lengths and sonicated in ethanol to remove any surface contaminants. EA was carried out in a custom-designed two-electrode electrochemical cell at room temperature using a DC power source (Keithley, Cleveland, OH, USA) with the current precisely monitored [29,30]. EA was performed using as-received Zr wire as the anode (5 mm exposed in the electrolyte) and non-targeted Zr wire as a cathode in an electrolyte with 1 M (NH_4_)_2_SO_4_ + 0.5 wt% NH_4_F. Anodization was performed at 20–100 V for 10–120 min, with current vs. time precisely recorded (Power Supply App, Keithley KickStart Software, Solon, OH, USA). Anodization voltage and time was decided based on current literature and prior optimizations studies using Ti wires [29]. Briefly, current density was calculated (current/area of anode) and plotted against time to visualize key features identifying anodization [29]. To remove the anodic oxide layer, anodized samples were sonicated in methanol for various time intervals to reveal the underlying features.

### 2.3. Surface Characterization

Surface topography characterization of the nanostructures was performed using scanning electron microscopy (JSM 7001F, JEOL, Tokyo, Japan). Before imaging, samples were mounted on an SEM holder using double-sided conductive tape and coated with a 5 nm thick layer of platinum. Images with a range of scan sizes at normal incidence and a 30° angle were acquired from the top surfaces.

## 3. Results and Discussion

Figure S1 (Supplementary Information) shows the SEM image of as-received Zr wire with clearly visible micro-machined features (micro-rough). There is an obvious resemblance to conventional dental implants/abutments with respect to the micro-scale features, which for dental implants, ensures osseointegration. This micro-roughness is regarded as the ‘gold-standard’ in dentistry and, hence, its removal to fabricate nanostructures could prove detrimental [31]. We have previously demonstrated that dual micro–nano features with nanopores superimposed on micro-machined Ti can be fabricated using an optimized EA procedure [32]. Fabrication of controlled nanostructures with preserved underlying micro-features on Zr implants can result in a paradigm shift in achieving enhanced bioactivity from nano-engineering, without compromising the benefits obtained from micro-roughness. In that light, we optimized the anodization of Zr implants using Zr wire as a model for Zr dental/orthopaedic implants with curved surfaces and micro-machined lines.

**Figure 2:**
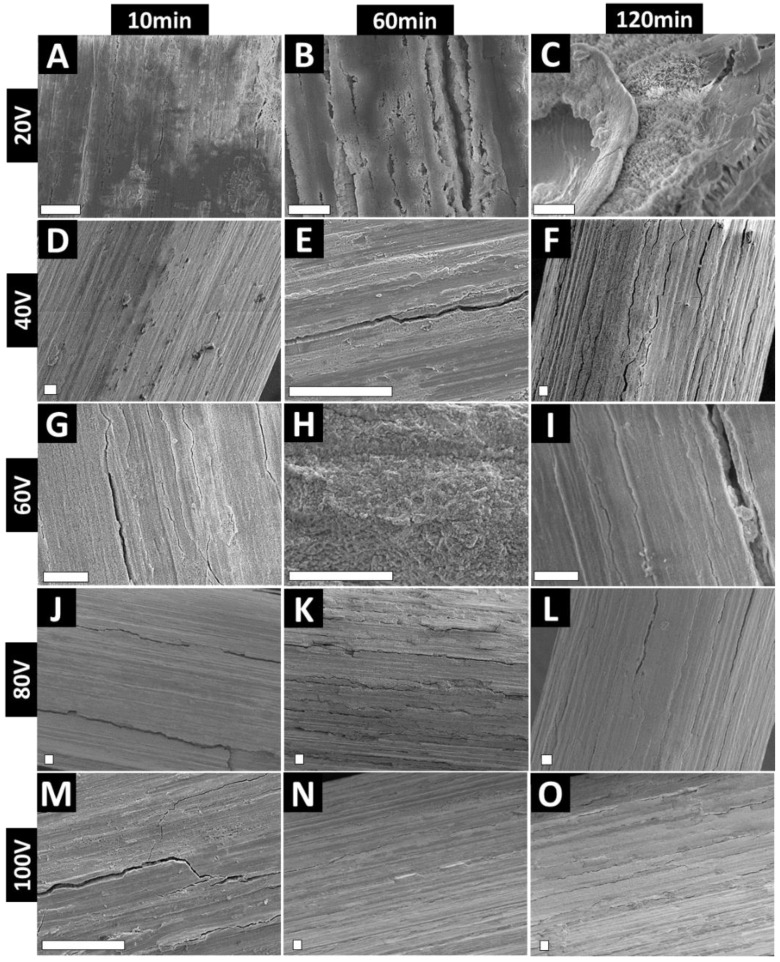
Top-view SEM images of anodized Zr wires at various voltages and times. (**A**–**C**) 20 V; (**D**–**F**) 40 V; (**G**–**I**) 60 V; (**J**–**L**) 80 V and (**M**–**O**) 100 V. Scale bars represent 20 μm.

Figure 2 shows low-magnification SEM images of the anodized wire, demonstrating an even coverage of the anodic ZrO_2_ film, with clearly visible cracks. We have previously reported similar cracks on TiO_2_ films formed on anodized Ti wire [32]. Briefly, these instabilities of the anodic layer could be attributed to the electric field concentrations at the topographical peaks of the substrate—which, in this case, is an irregular micro-rough curved surface [29,30]. The surface heterogeneity (micro-roughness) upon EA can also result in thicker oxide at the convex part and thinner oxide at the concave part [33]. It is noteworthy that these surface inconsistencies do not compromise the mechanical stability of the nano-engineered surface and can be used to accommodate drugs or enhance cellular adhesion [34]. Further, we have also explored strategies, including electrolyte ageing and surface polishing, to reduce anodic layer cracks [30].

**Figure 3:**
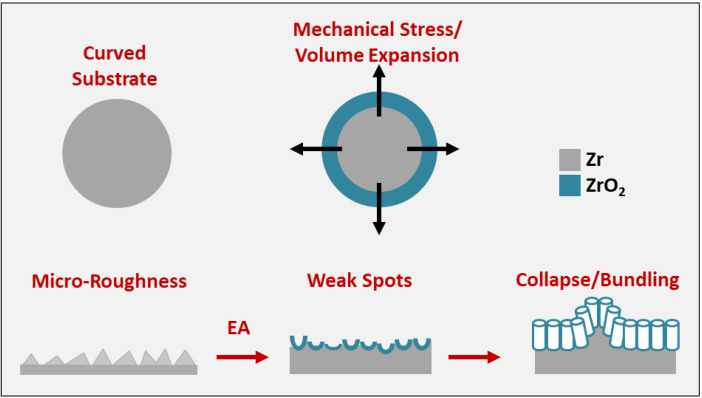
Representation of the formation of cracks or pits on the surface of anodized curved surfaces.

In summary, these cracks or pits are unavoidable on the anodized curved substrates and are attributed to (Figure 3):

(1) Curved substrate: radial/perpendicular growth of nanotubes outwards [29].
(2) Internal stresses: due to uneven electric field distribution.
(3) Mechanical stress: due to volume expansion and limited space for growth.
(4) Weak spots: electrolyte penetration resulting in unstable/fragile anodic layers [35].
(5) Substrate: micro-roughness further exacerbates the stresses/weak spots [36].
(6) Nanotube collapse (or bundling): especially for longer tubes.

Various strategies can be employed to reduce such cracks or instabilities on anodized metal surfaces (however, these remain poorly explored for Zr anodization) [37]:

(1) Use of appropriately aged electrolyte—mostly applicable to anodization with organic electrolytes (like ethylene glycol) [38,39].
(2) Polishing the substrate prior to anodization using mechanical, chemical or electropolishing treatments (will reduce/remove micro-roughness) [37].
(3) Reducing water content, voltage/current, or anodization time (may reduce diameter/length of anodized nanostructures due to reduced growth rates).

There have been attempts at exploring electrolyte ageing for Zr EA, with confirmation of the transition of nanoporous to nanotubular topography for EA performed in glycerol-based electrolyte [38,40]. It is noteworthy that such surface defects can also be reduced or minimized by electropolishing of the substrate, as shown elsewhere [29,41]. However, any polishing will ‘consume’ the underlying micro-roughness, removing this desirable feature of the implant and potentially compromising the positive osseointegrating property of the micro-roughness. Further, the cracks on the anodic film have been shown to survive drug loading and release in in vitro, ex vivo, and in vivo settings [34,42,43]. Indeed, cracks allow for higher drug loading amounts and enhance the overall surface roughness (at the microscale), allowing for higher cellular adhesion and anchoring points. Figure 2 also shows clear evidence of the anodic film with preserved micro-machined lines, with the anodic film aligned parallel to the lines on the underlying substrate. Cracks corresponding to voltage and time are also evident from Figure 2. Similar to Ti wire EA, cracks and instabilities increase with voltage and time of EA.

**Figure 4:**
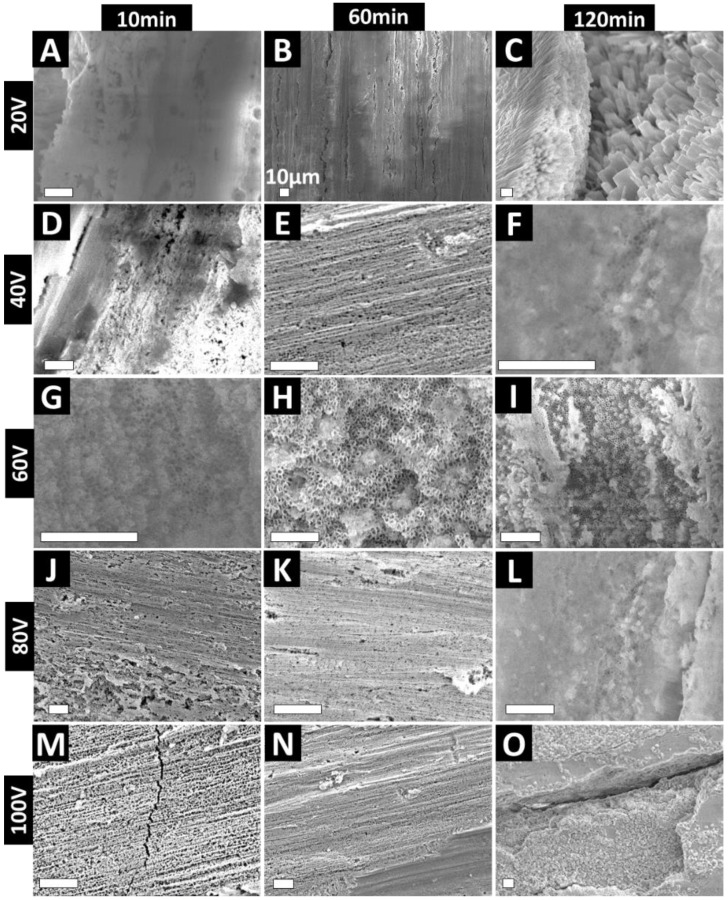
High-magnification SEM images showing various ZrO_2_ nanostructures formed on Zr wires at different voltage and times. (**A**–**C**) 20 V; (**D**–**F**) 40 V; (**G**–**I**) 60 V; (**J**–**L**) 80 V and (**M**–**O**) 100 V. Unmarked scale bars represent 1 μm.

High-magnification images of the anodized Zr wires are presented in Figure 4. At 20 V, a bare oxide layer with no distinguishable features is visible for 10–60 min of EA. For 120 min EA at 20 V, delamination of the oxide film reveals the presence of underlying nanocrystal-like features (Figure 4C). Using 40 V 60 min yielded alignment of the nanoporous layer onto the underlying micro-roughness (Figure 4E). However, for 40 V at 120 m, some evidence of the underlying nanotubular structures is visible, covered by the oxide film (Figure 4F). Further, clear evidence of nanopore formation is visible for 60 V at 10 m (diameter ~46 nm) and 60 m (diameter ~52 nm) (Figure 4G–I). In summary, for all of the 60 V anodized samples, we observed nanopore formation throughout the surface of the wire, with the irregular sponge-like patches of the ZrO_2_ layer (which was prominent for 60 V 10 m samples). It is worth noting that the nanopores on the Zr wire are aligned in the direction of the underlying microfeatures of the substrate. Our group has shown that aligned TiO_2_ nanopores on Ti can be used to mechanically stimulate cells [44,45]. Briefly, the activity of primary gingival fibroblasts and osteoblasts on aligned TiO_2_ nanopores was enhanced and the cells aligned parallel to the nanopores, indicating a strong mechanotransduction effect [45]. Additionally, as clear nanopores are visible, loading and release of various therapies may be enabled, which has never been demonstrated for ZrO_2_ nanopores and, hence, warrants further investigation. We have previously shown that that TiO_2_ nanopores are mechanically superior to conventional as well as mechanically enhanced (via various physical/chemical techniques) nanotubes (shown for TiO_2_ nanotubes) [37]. Additionally, for EA at 80–100 V for 10–60 m (Figure 4J–O), nanopore-like surface features were observed, which were aligned in the direction of the underlying substrate micro-roughness.

To elucidate the mechanism of formation of the various ZrO_2_ nanostructures, we undertook a detailed analysis of the current density (J) vs. time (t) plots, as presented in Figure S2 (Supplementary Information). The first 15 s of J vs. t plots provide information with respect to the first two phases of Zr EA: (1) formation of compact barrier layer (BL) and (2) pit formation [46]. There are significant differences between the J values at different voltages, and the presented data provide information about the time to reach equilibrium (teq) and barrier oxide layer (BL) thickness. The delay in reaching equilibrium equates to a thicker BL, strong adherence to the underlying substrate, and a stable anodic film, attributed to reduced compressive stress at the ZrO_2_–Zr interface [47]. teq and J are highest for the 60 V EA, which corresponds to a previous study showing that improved ordering is obtained for higher growth rates (or higher J values) [48]. This explains the findings from Figure 4G–I, which shows that the most nanoporous structures were obtained for 60 V EA. Based on J values corresponding to 60 V, an increased ‘outward expansion pressure’ for fast growth also explains the abovementioned. For EA performed at higher voltages (80 and 100 V), it can be assumed that the BL will be severely etched (higher field results in increased inward O^2−^ migration) and the electric field polarises the Zr–O bond and damages the tubular structures [46]. As previously reported, besides the internal growth-induced stresses, electric field-induced stresses can also result in compromised stability of the anodized ZrO_2_ film [29,32].

**Figure 5:**
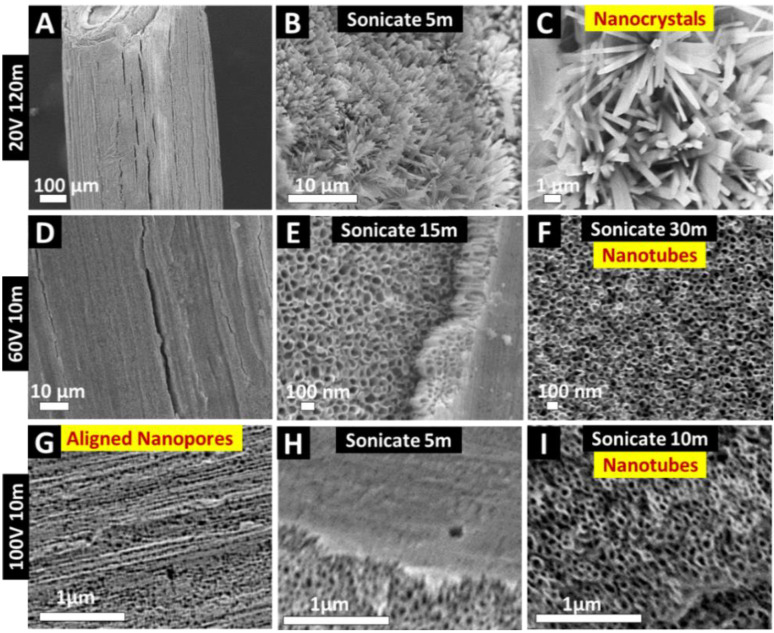
Top-view SEM images showing the influence of sonication of anodized Zr wire for various durations to remove superficial nanoporous oxide layer and expose underlying nanostructures. (**A**–**C**) 20 V 120 min anodized wire for 5 min sonication reveals nanocrystal-like features; (**D**–**F**) 15–30 min sonication of 60 V 10 min Zr wire reveals nanotubes; and (**G**–**I**) 5–10 min sonication removes oxide film and exposes underlying nanotubes on 100 V 10 min anodized wire. Survival of nanotubes on Zr wire post-sonication confirms mechanical stability and strong adherence to the underlying substrate.

Next, in order to expose the nanostructures covered by the ZrO_2_ film or nanopores, we sonicated the anodized wires at various times from 5–60 min. The resultant nanostructures are presented in Figure 5. It was found that dependent on the overall anodized film stability, higher sonication times disrupted the nanostructures. Five-minute sonication for the 20 V 120 min samples exposed the underlying nanocrystal-like topography, which was found to cover the underlying substrate (Figure 5B,C). For 60 V 10 m, 15 min sonication partially removed the nanoporous layer, while 30 m completely removed the nanopores, revealing the ZrO_2_ nanotubes (Figure 5E,F). The survival of the nanotubes even at 30 m sonication confirms the mechanical stability and robustness of the dual micro–nanostructures onto the underlying wire substrate. This correlates with previous studies whereby the microfeatures of the underlying substrates allowed for increased interfacial contact area between the anodic film and the substrate [35,40,41,42]. This increased area reduces the mechanical stress and volume expansion during anodic film growth and hence improves overall mechanical stability. Next, 10 min sonication of the aligned nanopores on 100 V 10 min wire revealed the ZrO_2_ nanotubes (Figure 5H,I) underneath. A similar effect was also observed for 80 V 10 m anodized wires, as shown in Figure S3 (Supplementary Information). It is noteworthy that for 60 V 10 m, the anodic structures survived the extended sonication time (15–30 m, Figure 5F), though for higher voltages (80 and 100 V, Figure 5I and Figure S3C), a small duration (5–10 min) exposed the underlying structures. We have previously shown that increased EA voltage is associated with higher growth rates on curved substrates, and hence reduced structural integrity of the anodized nanostructures (as compared to low-voltage-anodized structures) [29]. 

In summary, this study highlights the fabrication of stable nanotopographies on clinically relevant Zr surfaces—ensuring clinical translatability of electrochemically anodized Zr implants. The innovation of the study is the fact that it is a pioneering attempt at the fabrication of complex ZrO_2_ nanostructures on Zr curved surfaces via EA, while preserving the ‘gold standard’ micro-roughness to fabricate dual micro–nanostructures. Further, such controlled dual micro–nanostructures on Zr implants have the potential to augment cell activity and local therapy. Previous studies suggest that such aligned dual micro–nanostructures can mechanically stimulate cells [44]. Therefore, future studies will focus on the evaluation of soft- and hard-tissue integration on the surface of nano-engineered Zr implants, with the current study providing important data that bridges the gap to clinical translation by evaluating clinically relevant implant surfaces. It is noteworthy that bioactivity and therapeutic evaluations of such curved 3D implant substrates (Zr wires) is difficult to achieve in conventional 2D cell culture in vitro, which is more suitable for flat/planar substrates. We have previously undertaken extensive bioactivity evaluations of nano-engineered Ti wires in a 3D cell culture system in vitro [42], animal tissues ex vivo [43], and animal implantation in vivo [34]. However, inclusion of such detailed assessments is outside the scope of the current paper that is focussed on fabrication optimization.

## 4. Conclusions

With the objective of bridging the gap between nano-engineered zirconia and the dental implant industry, this study showcases the fabrication of various controlled nanotopographies on Zr wire substrates (as a model for dental implants) via electrochemical anodization (EA). In a pioneering approach, by tuning EA voltage and time, EA of micro-machined Zr wire enabled the fabrication of aligned nanopores, nanotubes, and nanocrystals. We also showed the impact of removing the top layer of oxide/nanopores to reveal the underlying nanotubes. Preserving the underlying micro-roughness and superimposition of controlled ZrO_2_ nanostructures holds great promise towards improving the bioactivity and therapeutic potential of conventional Zr-based dental and orthopaedic implants.

## Supplementary Materials

The following are available online at https://www.mdpi.com/article/10.3390/nano11040868/s1, Figure S1: SEM images of as-received Zr wires, Figure S2: current density vs. time plots for anodization of Zr wire, Figure S3: SEM images confirming the influence of sonication on 80 V 10 min anodized Zr wire.

## References

[1] Zhang Y., Chen H.-X., Duan L., Fan J.-B. (2021). The Electronic Structures, Elastic Constants, Dielectric Permittivity, Phonon Spectra, Thermal Properties and Optical Response of Monolayer Zirconium Dioxide: A First-Principles Study. Thin Solid Film..

[2] Schünemann F.H., Galárraga-Vinueza M.E., Magini R., Fredel M., Silva F., Souza J.C.M., Zhang Y., Henriques B. (2019). Zirconia Surface Modifications for Implant Dentistry. Mater. Sci. Eng. C.

[3] Rupp F., Liang L., Geis-Gerstorfer J., Scheideler L., Hüttig F. (2018). Surface Characteristics of Dental Implants: A Review. Dent. Mater..

[4] Guo T., Gulati K., Arora H., Han P., Fournier B., Ivanovski S. (2021). Race to Invade: Understanding Soft Tissue Integration at the Transmucosal Region of Titanium Dental Implants. Dent. Mater..

[5] Tuna T., Wein M., Swain M., Fischer J., Att W. (2015). Influence of Ultraviolet Photofunctionalization on the Surface Characteristics of Zirconia-Based Dental Implant Materials. Dent. Mater..

[6] Honda J., Komine F., Kusaba K., Kitani J., Matsushima K., Matsumura H. (2020). Fracture Loads of Screw-Retained Implant-Supported Zirconia Prostheses after Thermal and Mechanical Stress. J. Prosthodont. Res..

[7] AlFarraj A.A., Aldosari A., Sukumaran A., Al Amri M.D., van Oirschot A.J.A.B., Jansen J.A. (2018). A Comparative Study of the Bone Contact to Zirconium and Titanium Implants after 8 Weeks of Implantation in Rabbit Femoral Condyles. Odontology.

[8] Sivaraman K., Chopra A., Narayan A.I., Balakrishnan D. (2018). Is Zirconia a Viable Alternative to Titanium for Oral Implant? A Critical Review. J. Prosthodont. Res..

[9] Patil N.A., Kandasubramanian B. (2020). Biological and mechanical enhancement of zirconium dioxide for medical applications. Ceram. Int..

[10] Gomez S.A., Schreiner W., Duffó G., Ceré A.S. (2011). Surface Characterization of Anodized Zirconium for Biomedical Applications. Appl. Surf. Sci..

[11] Wang Y.B., Zheng Y.F., Wei S.C., Li M. (2011). In Vitro Study on Zr-Based Bulk Metallic Glasses as Potential Biomaterials. J. Biomed. Mater. Res. Part B Appl. Biomater..

[12] Hobbs L.W., Rosen V.B., Mangin S.P., Treska M., Hunter G. (2005). Oxidation Microstructures and Interfaces in the Oxidized Zirconium Knee. Int. J. Appl. Ceram. Technol..

[13] Uchida M., Kim H.M., Miyaji F., Kokubo T., Nakamura T. (2002). Apatite Formation on Zirconium Metal Treated with Aqueous Naoh. Biomaterials.

[14] Gomez S.A., Ballarre J., Orellano J.C., Duffó G., Cere S. (2013). Surface Modification of Zirconium by Anodisation as Material for Permanent Implants: In Vitro and in Vivo Study. J. Mater. Sci. Mater. Med..

[15] Jović V.D., Jović B.M. (2008). The Influence of the Conditions of the ZrO_2_ Passive Film Formation on Its Properties in 1 M NaOH. Corros. Sci..

[16] Gulati K., Hamlet S.M., Ivanovski S. (2018). Tailoring the Immuno-Responsiveness of Anodized Nano-Engineered Titanium Implants. J. Mater. Chem. B.

[17] de la Hoz M.F.T., Katunar M.R., González A., Sanchez A.G., Díaz A.O., Ceré S. (2019). Effect of Anodized Zirconium Implants on Early Osseointegration Process in Adult Rats: A Histological and Histomorphometric Study. Prog. Biomater..

[18] Bacchelli B., Giavaresi G., Franchi M., Martini D., de Pasquale V., Trirè A., Fini M., Giardino R., Ruggeri A. (2009). Influence of a Zirconia Sandblasting Treated Surface on Peri-Implant Bone Healing: An Experimental Study in Sheep. Acta Biomater..

[19] Zhang L., Zhu S., Han Y., Xiao C., Tang W. (2014). Formation and Bioactivity of Ha Nanorods on Micro-Arc Oxidized Zirconium. Mater. Sci. Eng. C.

[20] Quan R., Yang D., Yan J., Li W., Wu X., Wang H. (2009). Preparation of Graded Zirconia–Cap Composite and Studies of Its Effects on Rat Osteoblast Cells in Vitro. Mater. Sci. Eng. C.

[21] Li X., Deng J., Lu Y., Zhang L., Sun J., Wu F. (2019). Tribological Behavior of ZrO_2_/Ws_2_ Coating Surfaces with Biomimetic Shark-Skin Structure. Ceram. Int..

[22] Gulati K., Kogawa M., Maher S., Atkins G., Findlay D., Losic D. (2015). Titania Nanotubes for Local Drug Delivery from Implant Surfaces. Electrochemically Engineered Nanoporous Materials.

[23] Tsuchiya H., Macak j., Taveira L., Schmuki P. (2005). Fabrication and Characterization of Smooth High Aspect Ratio Zirconia Nanotubes. Chem. Phys. Lett..

[24] Katunar M.R., Sanchez A.G., Coquillat A.S., Civantos A., Campos E.M., Ballarre J., Vico T., Baca M., Ramos V., Cere S. (2017). In vitro and in vivo characterization of anodised zirconium as a potential material for biomedical applications. Mater. Sci. Eng. C.

[25] Zhao J., Xu R., Wang X., Li Y. (2008). In Situ Synthesis of Zirconia Nanotube Crystallines by Direct Anodization. Corros. Sci..

[26] Guo L., Zhao J., Wang X., Xu R., Lu Z., Li Y. (2009). Bioactivity of Zirconia Nanotube Arrays Fabricated by Electrochemical Anodization. Mater. Sci. Eng. C.

[27] Frandsen C.J., Brammer K.S., Noh K., Connelly L.S., Oh S., Chen L.H., Jin S. (2011). Zirconium Oxide Nanotube Surface Prompts Increased Osteoblast Functionality and Mineralization. Mater. Sci. Eng. C.

[28] Zhang L., Han Y. (2011). Enhanced Bioactivity of Self-Organized ZrO_2_ Nanotube Layer by Annealing and Uv Irradiation. Mater. Sci. Eng. C.

[29] Gulati K., Santos A., Findlay D., Losic D. (2015). Optimizing Anodization Conditions for the Growth of Titania Nanotubes on Curved Surfaces. J. Phys. Chem. C.

[30] Gulati K., Maher S., Chandrasekaran S., Findlay D.M., Losic D. (2016). Conversion of Titania (TiO_2_) into Conductive Titanium (Ti) Nanotube Arrays for Combined Drug-Delivery and Electrical Stimulation Therapy. J. Mater. Chem. B.

[31] Gulati K., Ivanovski S. (2017). Dental Implants Modified with Drug Releasing Titania Nanotubes: Therapeutic Potential and Developmental Challenges. Expert Opin. Drug Deliv..

[32] Gulati K., Li T., Ivanovski S. (2018). Consume or Conserve: Microroughness of Titanium Implants toward Fabrication of Dual Micro–Nanotopography. ACS Biomater. Sci. Eng..

[33] Zhao J., Wang X., Xu R., Meng F., Guo L., Li Y. (2008). Fabrication of High Aspect Ratio Zirconia Nanotube Arrays by Anodization of Zirconium Foils. Mater. Lett..

[34] Kaur G., Willsmore T., Gulati K., Zinonos I., Wang Y., Kurian M., Hay S., Losic D., Evdokiou A. (2016). Titanium Wire Implants with Nanotube Arrays: A Study Model for Localized Cancer Treatment. Biomaterials.

[35] Proost J., Vanhumbeeck J., van Overmeere Q. (2009). Instability of Anodically Formed TiO_2_ Layers (Revisited). Electrochim. Acta.

[36] Fan M., la Mantia F. (2013). Effect of Surface Topography on the Anodization of Titanium. Electrochem. Commun..

[37] Li T., Gulati K., Wang N., Zhang Z., Ivanovski S. (2018). Understanding and Augmenting the Stability of Therapeutic Nanotubes on Anodized Titanium Implants. Mater. Sci. Eng. C.

[38] Muratore F., Hashimoto T., Skeldon P., Thompson G.E. (2011). Effect of Ageing in the Electrolyte and Water on Porous Anodic Films on Zirconium. Corros. Sci..

[39] Guo T., Oztug N.A.K., Han P., Ivanovski S., Gulati K. (2021). Old Is Gold: Electrolyte Aging Influences the Topography, Chemistry, and Bioactivity of Anodized TiO_2_ Nanopores. ACS Appl. Mater. Interfaces.

[40] Li T., Gulati K., Wang N., Zhang Z., Ivanovski S. (2018). Bridging the Gap: Optimized Fabrication of Robust Titania Nanostructures on Complex Implant Geometries Towards Clinical Translation. J. Colloid Interface Sci..

[41] Pilling N.B. (1923). The Oxidation of Metals at High Temperature. J. Inst. Met..

[42] Gulati K., Kogawa M., Prideaux M., Findlay D.M., Atkins G.J., Losic D. (2016). Drug-Releasing Nano-Engineered Titanium Implants: Therapeutic Efficacy in 3D Cell Culture Model, Controlled Release and Stability. Mater. Sci. Eng. C.

[43] Rahman S., Gulati K., Kogawa M., Atkins G.J., Pivonka P., Findlay D.M., Losic D. (2016). Drug Diffusion, Integration, and Stability of Nanoengineered Drug-Releasing Implants in Bone Ex-Vivo. J. Biomed. Mater. Res. Part A.

[44] Gulati K., Moon H.G., Kumar P.T.S., Han P., Ivanovski S. (2020). Anodized Anisotropic Titanium Surfaces for Enhanced Guidance of Gingival Fibroblasts. Mater. Sci. Eng. C.

[45] Gulati K., Moon H.G., Li T., Kumar P.T.S., Ivanovski S. (2018). Titania Nanopores with Dual Micro-/Nano-Topography for Selective Cellular Bioactivity. Mater. Sci. Eng. C.

[46] Ismail S., Ahmad Z.A., Berenov A., Lockman Z. (2011). Effect of Applied Voltage and Fluoride Ion Content on the Formation of Zirconia Nanotube Arrays by Anodic Oxidation of Zirconium. Corros. Sci..

[47] Zhou X., Nguyen N.T., Özkan S., Schmuki P. (2014). Anodic Tio_2_ Nanotube Layers: Why Does Self-Organized Growth Occur—A Mini Review. Electrochem. Commun..

[48] Jessensky O., Müller F., Gösele U. (1998). Self-Organized Formation of Hexagonal Pore Arrays in Anodic Alumina. Appl. Phys. Lett..

