# Supplementary material for: Towards Clinical Translation: Optimized Fabrication of Controlled Nanostructures on Implant-Relevant Curved Zirconium Surfaces
Source: Nanomaterials (Basel). 2021 Mar 29;11(4):868. doi: 10.3390/nano11040868 (PMC8067134; doi:10.3390/nano11040868)
Supplement: Supplementary file 1 [file nanomaterials-11-00868-s001.pdf]

Supplementary Materials

Towards Clinical Translation: Optimized Fabrication of Controlled Nanostructures on Implant-Relevant Curved Zirconium Surfaces

Divya Chopra, Karan Gulati * and Sašo Ivanovski *

The University of Queensland, School of Dentistry, Herston QLD 4006, Australia; d.chopra@uq.net.au
* Correspondence: k.gulati@uq.edu.au (K.G.); s.ivanovski@uq.edu.au (S.I.)

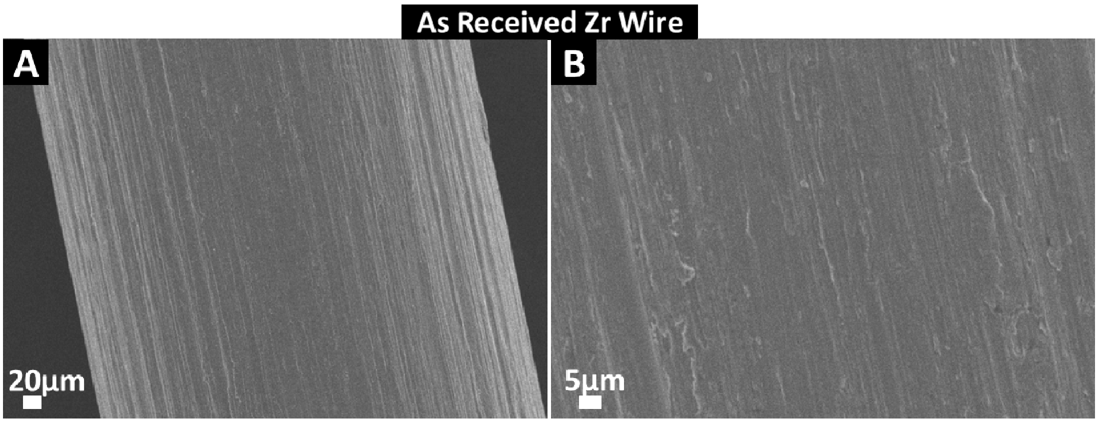

Figure S1. Top-view SEM images: (**A**,**B**) as-received 'micro-rough' Zr wire showing micro-machined lines, which resembles clinically utilized implant surfaces.

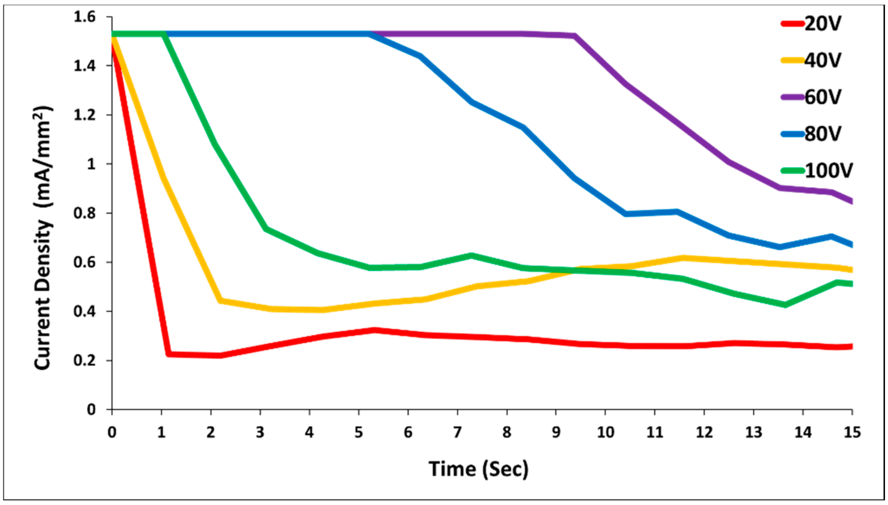

Figure S2. Current density (J) vs. time plots for anodization performed on Zr wire at different voltages for 15 s. The anodization was performed for 10 min, but the plot only shows first 15 s that define the formation of specific nanostructures.

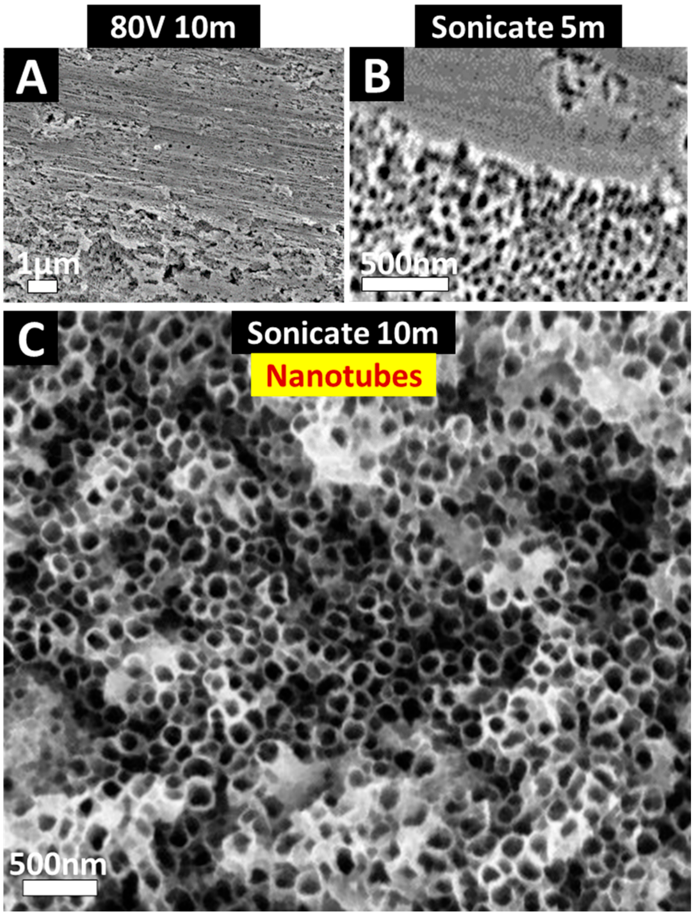

Figure S3. Top-view SEM Images showing effect of sonication on (**A**) anodized Zr wire (80 V 10 min) to expose underlying nanotubes (**B**,**C**).
